# GATA binding protein 6 (*GATA6*) is co-amplified with *PIK3CA* in patients with esophageal adenocarcinoma and is linked to neoadjuvant therapy

**DOI:** 10.1007/s00432-020-03486-2

**Published:** 2020-12-10

**Authors:** Patrick Sven Plum, Heike Löser, Thomas Zander, Ahlem Essakly, Christiane J. Bruns, Axel M. Hillmer, Hakan Alakus, Wolfgang Schröder, Reinhard Büttner, Florian Gebauer, Alexander Quaas

**Affiliations:** 1 Department of General, Visceral, Cancer, and Transplantation Surgery, University of Cologne, Faculty of Medicine, and University Hospital Cologne, Kerpener Straße 62, 50937 Cologne, Germany; 2 Gastrointestinal Cancer Group Cologne (GCGC), Cologne, Germany; 3 Else Kröner Forschungskolleg Cologne “Clonal Evolution in Cancer”, Cologne, Germany; 4 Centre for Integrated Oncology (CIO), Cologne Bonn, Cologne, Germany; 5 Institute of Pathology, University of Cologne, Faculty of Medicine, and University Hospital Cologne, Kerpener Straße 62, 50937 Cologne, Germany; 6 Department of Internal Medicine I, University of Cologne, Faculty of Medicine, and University Hospital Cologne, Kerpener Straße 62, 50937 Cologne, Germany

**Keywords:** GATA6, PIK3CA, Esophageal adenocarcinoma, EAC, Prognosis, Biomarker, Neoadjuvant therapy, Treatment response, Neoadjuvant treatment

## Abstract

**Purpose:**

Driver mutations are typically absent in esophageal adenocarcinoma (EAC). Mostly, oncogenes are amplified as driving molecular events (including GATA6-amplification in 14% of cases). However, only little is known about its biological function and clinical relevance.

**Methods:**

We examined a large number of EAC (*n* = 496) for their *GATA6* amplification by fluorescence in situ hybridization (FISH) analyzing both primary resected (*n* = 219) and neoadjuvant treated EAC (*n* = 277). Results were correlated to clinicopathological data and known mutations/amplifications in our EAC-cohort.

**Results:**

*GATA6* amplification was detectable in 49 (9.9%) EACs of our cohort. We observed an enrichment of *GATA6*-positive tumors among patients after neoadjuvant treatment (12,3% amplified tumors versus 6,8% in the primary resected group; *p* = 0.044). Additionally, there was a simultaneous amplification of *PIK3CA* and *GATA6* (*p* < 0.001) not detectable when analyzing other genes such as *EGFR*, *ERBB2, KRAS* or *MDM2*. Although we did not identify a survival difference depending on *GATA6* in the entire cohort (*p* = 0.212), *GATA6* amplification was associated with prolonged overall survival among patients with primary surgery (median overall-survival 121.1 vs. 41.4 months, *p* = 0.032). Multivariate cox-regression analysis did not confirm *GATA6* as an independent prognostic marker, neither in the entire cohort (*p* = 0.210), nor in the subgroup with (*p* = 0.655) or without pretreatment (*p* = 0.961).

**Conclusions:**

Our study investigates the relevance of *GATA6* amplification on a large tumor collective, which includes primary resected tumors and the clinically relevant group of neoadjuvant treated EACs. Especially in the pretreated group, we found an accumulation of *GATA6*-amplified tumors (12.3%) and a frequent co-amplification of *PIK3CA*. Our data suggest an increased resistance to radio-chemotherapy in *GATA6*-amplified tumors.

## Introduction

Even today, esophageal adenocarcinoma (EAC) is a devastating gastrointestinal malignancy with an overall five-years survival ranging from 15 to 20% (DeSantis et al. 2014; Rustgi and El-Serag 2014; Coleman et al. 2018) and still increasing incidences (Arnold et al. 2017). In the recent past, efforts focused on developing more effective multimodal treatment concepts including neoadjuvant chemoradiation or perioperative chemotherapy (Al-Batran et al. 2008; van Hagen et al. 2012). Therapeutic decisions are based on mere clinical parameters deriving from staging examinations and success of neoadjuvant therapy is evaluated depending on the degree of therapeutic response towards this treatment (Shapiro et al. 2015; Al-Batran et al. 2019). However, not all patients benefit from this still very standardized treatment routines, developing only significant toxic side effects. This is the case in 35% of patients undergoing chemoradiation and 39% of patients under chemotherapy (Ronellenfitsch et al. 2016; den Bakker et al. 2017). This clinical dilemma is due to the fact that EAC is a genetic extremely heterogenous disease. Its mutational burden is enormous (Mourikis et al. 2019; von Loga et al. 2020) and EAC is often associated with a high chromosomal instability (Frankell et al. 2019). Whole exome sequencing revealed *TP53, CDKN2A, SMAD4, ARID1A, VEGFA, CCNE1* and *PIK3CA* to be among those genes most frequently affected (Dulak et al. 2013; Cancer Genome Atlas Research Network et al. 2017)*.* Another common phenomenon is the principle of genetical amplifications [also known as copy number alterations (CNAs)]. According to recent data analyzing the genetic landscape of 551 EACs these amplifications mostly occur in *KRAS* (19%), *c-MYC* (19%), *HER2* (18%), *CCND1* (14%) and *GATA6* (14%) (Frankell et al. 2019). However, only little is known about the function of *GATA6* amplification within this entity.

GATA binding protein 6 (*GATA6*) belongs to the GATA family comprising of the members GATA1-6 and its gene is located on chromosome 18 (q11.1 ~ q11.2) within the human genome (Suzuki et al. 1996). During embryogenesis, *GATA6* is highly expressed within the endoderm and mesoderm (Carrasco et al. 2012) as it is essential for the development of different tissues such as adrenal gland and the central nervous system (Jimenez et al. 2003; Kamnasaran and Guha 2005). Being a transcriptional factor, dysregulation of *GATA6* can also result in pathological changes and it was demonstrated that *GATA6* alterations implicated in several malignancies such as non-small lung cancer (NSCLC), gastric cancer, cholangiocarcinoma, pancreatic adenocarcinoma or colorectal adenocarcinoma (Zhong et al. 2011; Shen et al. 2013; Tian et al. 2013; Van Baal et al. 2013; Ma et al. 2019). For esophageal adenocarcinoma, it has been shown in a study including 85 EACs that gene amplification of *GATA6* affected the patients’ survival in a negative manner (Lin et al. 2012). During the development of Barrett’s esophagus and the following malignant transformation, the expression of *GATA6* is successively increasing resembling its impact on the progression of the disease (Pavlov et al. 2015).

Aim of the current study was to analyze the relevance and frequency of *GATA6* amplification in a large cohort of EAC patients and the consecutively correlation with clinical, pathological and molecular parameters as well as the patients’ survival.

## Materials and methods

### Patients and tumor samples

Analysis was performed on 496 patients with esophageal adenocarcinoma who either underwent primary surgical resection or resection after neoadjuvant treatment between 1999 and 2017 at the Department of General, Visceral, Cancer and Transplant Surgery, University of Cologne, Germany. All patients underwent primary staging including contrast-enhanced computed tomography, esophagoduodenoscopy, endoscopic ultrasound and physical examination. Patients who qualified for multimodal treatment because of locally advanced tumors (cT > 2) or suspected locoregional lymph node metastases (cN +) received neoadjuvant chemoradiation (van Hagen et al. 2012) or chemotherapy (Donohoe and Reynolds 2017). The standardized surgical procedure was transthoracic en-bloc esophagectomy with two-field lymphadenectomy of the abdominal and mediastinal lymph nodes, reconstruction via gastric pull-up and intrathoracic anastomosis (Ivor-Lewis esophagectomy). The abdominal part was predominantly performed via laparoscopy while thoracotomy was open surgery (hybrid esophagectomy). For more technical details we refer to previous publications (Plum et al. 2018) and other authors (Mariette et al. 2019). Informed consent and ethical approval were obtained from all participating patients. This retrospective study was performed according to the criteria of the ethics committee of the University Hospital of Cologne (No. 13–091 and 10–242) and in accordance with the relevant version of the Helsinki Declaration. Clinical data was collected prospectively within the department according to a standardized protocol. During the first two years, clinical follow-up of patients was performed in the out-patient clinics every three months, followed by annual exams. These included clinical evaluation, abdominal ultrasound, chest X-ray and additional diagnostic procedures as required.

Single-spot tissue microarrays (TMA) were constructed from all surgical specimens for fluorescence in-situ hybridization (FISH) and immunohistochemical analysis. The exact procedure has been described before (Simon et al. 2005; Helbig et al. 2016). In principle, tissue cylinders with a diameter of 1.2 mm each were punched from the selected tumor tissue blocks (donor blocks) via a self-constructed semi-automated precision instrument and embedded on an empty paraffin block (recipient block). Four µm sections of the resulting TMA blocks were transferred to an adhesive coated slide system (Instrumedics Inc., Hackensack, NJ, USA) for following FISH or immunohistochemistry. Amplification of *GATA6* (via FISH) was correlated with molecular profiles of these EAC samples including assessments of *ARIDA 1A* loss*, TP53* mutations as well as ERBB2, c-MYC, *KRAS* and *PIK3CA* amplifications.

### Fluorescence in-situ hybridization (FISH) of *GATA6*

Fluorescence in-situ hybridization (FISH) analysis for the evaluation of *GATA6* gene copy numbers was performed with GATA6-20-GR Probe (Empire Genomics, New York, NY, USA) and the Zytolight centromere 18 (CEN18) Probe (Zytovision Bremerhaven, Germany) on the resulting TMA slides. For PIK3CA gene amplification analysis, the Zytolight SPEC PIK3CA/CEN3 Dual Probe Kit (Zytovision, Germany) was used according to the manufacturers' protocol. Three µm tissue sections on slides (SuperFrost Plus) were mounted by heating, followed by deparaffinization, protease digestion, washing steps (VP2000 processor system, Abbott Molecular, Wiesbaden, Germany) and hybridization at 37 °C overnight with the FISH Probe. The slides were stained with DAPI before analysis. Cases were further evaluated only when normal tissue nuclei displayed one or two clearly distinct signals of green *GATA6* and orange CEN18. Tumor tissue was scanned for amplification hot spots of *GATA6* signals using × 63 objective (DM5500 fluorescent microscope; Leica). This reading strategy followed that of the c-MYC-FISH probe to evaluate areas of cluster amplification. GATA6 amplification was defined as gene copy cluster > 50% of the tumor cells, respectively, gene copy number > 6 per cell. For PIK3CA reading strategy followed the recommendations of previous studies amplification such as (Essakly et al. 2020).

### Immunohistochemistry

Immunohistochemistry (IHC) was performed on TMA slides using the following antibodies against MHC1, PDL1, LAG3, IDO, INI, VISTA, TP53, TIM3, TUBB3, HER2, Ki67, ARIDA 1A, BRG1, BRM, Met1 and c-MYC as already published by our group (Becker et al. 2015; Loeser et al. 2019; Plum et al. 2019; Essakly et al. 2020; Gebauer et al. 2020; Wagener-Ryczek et al. 2020; Schiffmann et al. 2020).

### Statistical analysis

SPSS Statistics for Mac (Version 21, SPSS) was used for statistical analysis. Interdependence between stainings and clinical data were calculated using the chi-squared and Fisher’s exact tests, and displayed by cross-tables. Survival curves were plotted using the Kaplan–Meier method and analyzed using the log-rank test. All tests were two-sided. *p* values < 0.05 were considered statistically significant.

## Results

### Patients’ baseline characteristics

A total of 496 patients of 685 on the TMA with EAC were interpretable on the single-spot for *GATA6*. Reasons for non-informative cases (189 spots; 27.6%) included lack of tissue samples or absence of unequivocal cancer tissue in the TMA spot. Clinico-pathological data were summarized within Table 1. The majority of patients were male (male: *n* = 437; 88.1% versus female: *n* = 59; 11.9%). The median age was 65.2 years (range 33.6–85.6 years) at the time point of diagnosis. More than half of the patient cohort (*n* = 277; 55.8%) underwent multimodal treatment (including either chemoradiation or chemotherapy before surgical resection) while 219 (44.2%) patients received primary surgery.

**Table 1:** Clinico-pathological parameters for the patient cohort

Factor	Total		GATA6				*p* value
			Negative		Positive		
Sex							
Female	59	11.9%	53	89.8%	6	10.2%	
Male	437	88.1%	394	90.2%	43	9.8%	0.937
Agegroup							
< 65 yrs	245	52,5%	216	88.2%	29	11.8%	
> 65 yrs	222	47.5%	202	91.0%	20	9.0%	0.319
Tumor stage							
pT1/2	125	25.4%	112	89.6%	13	10.4%	
pT3/4	368	74.6%	332	90.2%	36	9.8%	0.842
Lymph node metastasis							
pN0	198	40.1%	178	89.9%	20	4.0%	
pN +	296	59.9%	267	90.2%	29	9.8%	0.912
Grading							
G1	5	1.4%	5	1.4%	0	0%	
G2	197	55.5%	178	90.4%	19	9.6%	
G3	151	42.5%	137	90.7%	14	9.3%	
G4	2	0.6%	2	0.6%	0	0%	0.746
UICC							
I	108	22.0%	97	89.8%	11	10.2%	
II	106	21.5%	96	90.6%	10	9.4%	
III	208	42.3%	184	88.5%	24	11.5%	
IV	70	14.2%	66	94.3%	4	5.7%	0.567
Neoadjuvant therapy							
No	219	44.2%	204	93.2%	15	6.8%	
Yes	277	55.8%	243	87.7%	34	12.3%	0.044

### *GATA6* amplification in esophageal adenocarcinoma and correlation to clinico-pathological data

**Fig. 1:**
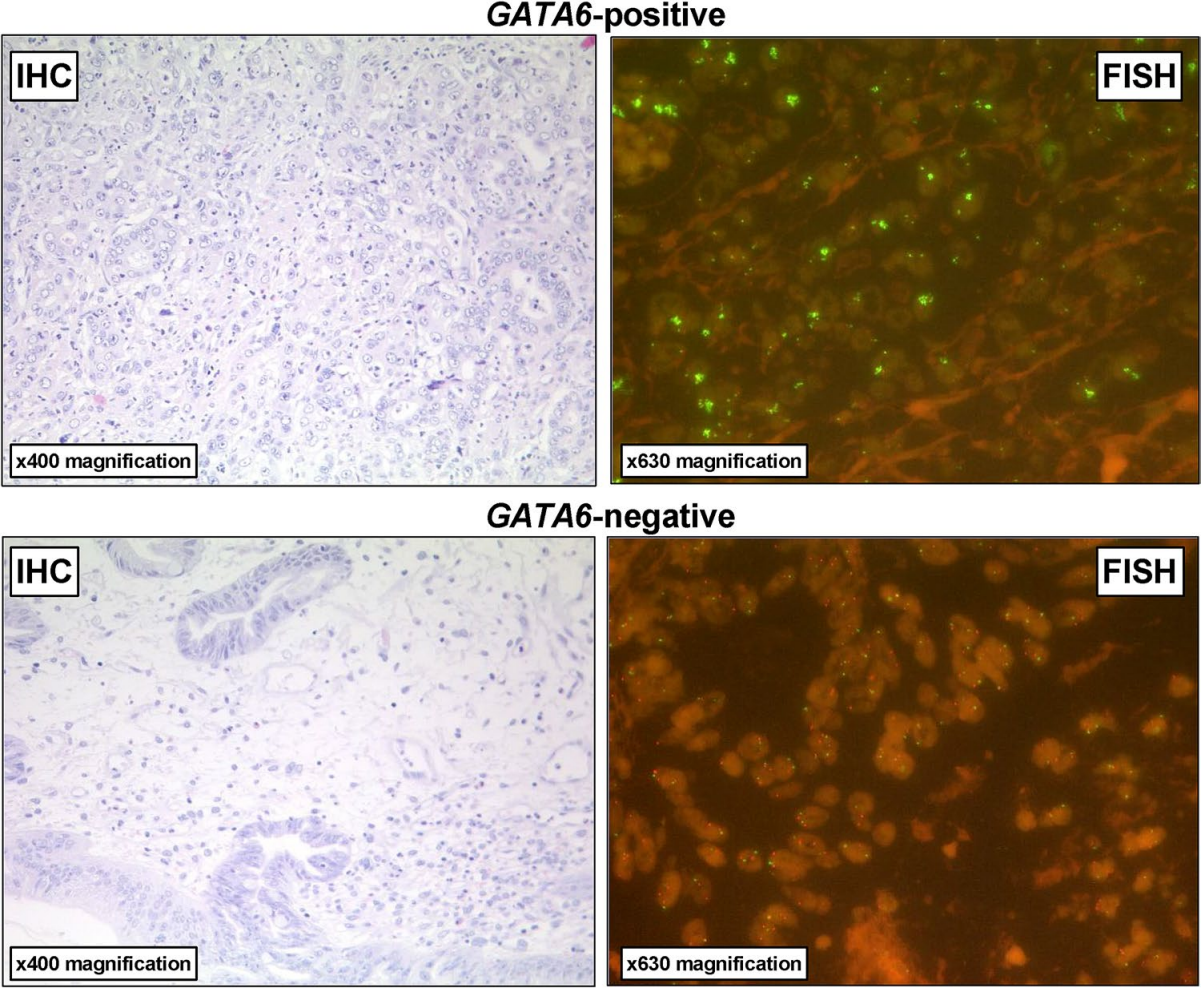
Representative images of immunohistochemistry (IHC) and fluorescence in-situ hybridization (FISH) analysis for the evaluation of *GATA6* gene copy numbers using the GATA6-20-GR (green) and the Zytolight centromere 18 (CEN18) (red) Probe illustrating (upper row) GATA6-positive versus (lower row) *GATA6*-negative esophageal adenocarcinoma. *GATA6* amplification was defined as gene copy cluster > 50% of the tumor cells, respectively, gene copy number > 6 per cell

Considering the entire patient cohort, *GATA6* amplification was detectable via FISH in 49 patients (9.9%) within an intranuclear pattern (compare Fig. 1). There was no significant correlation between such clinico-pathological parameters such as sex, age, grading, (y)pT-category, (y)pN-category or UICC-stage (see Table 1). However, *GATA6* amplification was correlated with the status of neoadjuvant treatment (*p* = 0.044). Patients who had multimodal therapy showed in 12.3% an amplification in the FISH examination compared to 6.8% among those patients who had primary esophagectomy.

### *GATA6* and *PIK3CA* co-amplification

FISH-data of *GATA6* amplification was additionally correlated with other important biomarkers in EAC like other amplified oncogenes, immune checkpoint markers such as *PD-L1, LAG3, IDO, INI*, *VISTA or* the antigen-presenting protein MHC1*,* as well as additional proteins like the chromatin-remodeler and SWI/SNF components *ARIDA 1A, BRG1, BRM* and oncogene amplifications like *MET, c-MYC, KRAS, ERBB2, MDM2* and *PIK3CA*. We observed no correlation between *GATA6* and most of these other biomarkers within the cohort performing the cross-table analysis (see Table 2). However, we identified co-amplification of *GATA6* together with *PIK3CA* in 9 (1.8%) patients of the entire cohort (*p* < 0.001) divided into 2 (0.3%) patients of the pretreated subgroup (*p* < 0.001) and 7 (1.4%) patients with primary surgery (*p* = 0.174). PIK3CA amplifications were seen in 24 patients (4.8%) (Essakly et al. 2020). Similar amplification rates were seen within the primary surgery group (*n* = 11; 5.0%) and surgery after neoadjuvant treatment (*n* = 13; 4.7%). All details are illustrated in Table 3.

**Table 2:** Correlation between *GATA6* and other molecular markers within the patient cohort

Factor	Total		GATA6				*p* value
			Negative		Positive		
HER2							
Normal	300	87.7%	268	89.3%	32	10.7%	
Mutated	42	12.3%	41	97.6%	1	2.4%	0.089
MHC1							
Loss	106	29.4%	101	95.3%	5	4.7%	
Normal	254	70.6%	227	89.4%	27	10.6%	0.072
ARIDA 1A							
Loss	45	9.5%	44	97.8%	1	2.2%	
Normal	427	90.5%	381	89.2%	46	10.8%	0.068
C-myc							
Normal	418	87.8%	379	90.7%	39	9.3%	
Amplified	58	12.2%	48	82.8%	10	17.2%	0.063
KRAS							
Normal	402	82.4%	366	91.0%	36	9.0%	
Mutated	86	17.6%	73	84.9%	13	15.1%	0.084
PIK3CA							
Normal	415	94.5%	379	91.3%	36	8.7%	
Mutated	24	5.5%	15	62.5%	9	37.5%	< 0.001

**Table 3:** Correlation between *GATA6* and *PIK3CA* within the patient cohort

Factor		Total		GATA6				*p* value
				Negative		Positive		
Entire cohort								
PIK3CA	Normal	415	94.5%	379	91.3%	36	8.7%	
	Amplified	24	5.5%	15	62.5%	9	37.5%	< 0.001
Patients without neoadjuvant treatment								
PIK3CA	Normal	186	94.4%	173	93.0%	13	7.0%	
	Amplified	11	5.6%	9	81.8%	2	18.2%	0.174
Patients with neaodjuvant treatment								
PIK3CA	Normal	229	94.6%	206	90.0%	23	10.0%	
	Amplified	13	5.4%	6	46.2%	7	53.8%	< 0.001

### *GATA6* amplification is associated with a prolonged survival among patients who did not receive neoadjuvant treatment

**Fig. 2:**
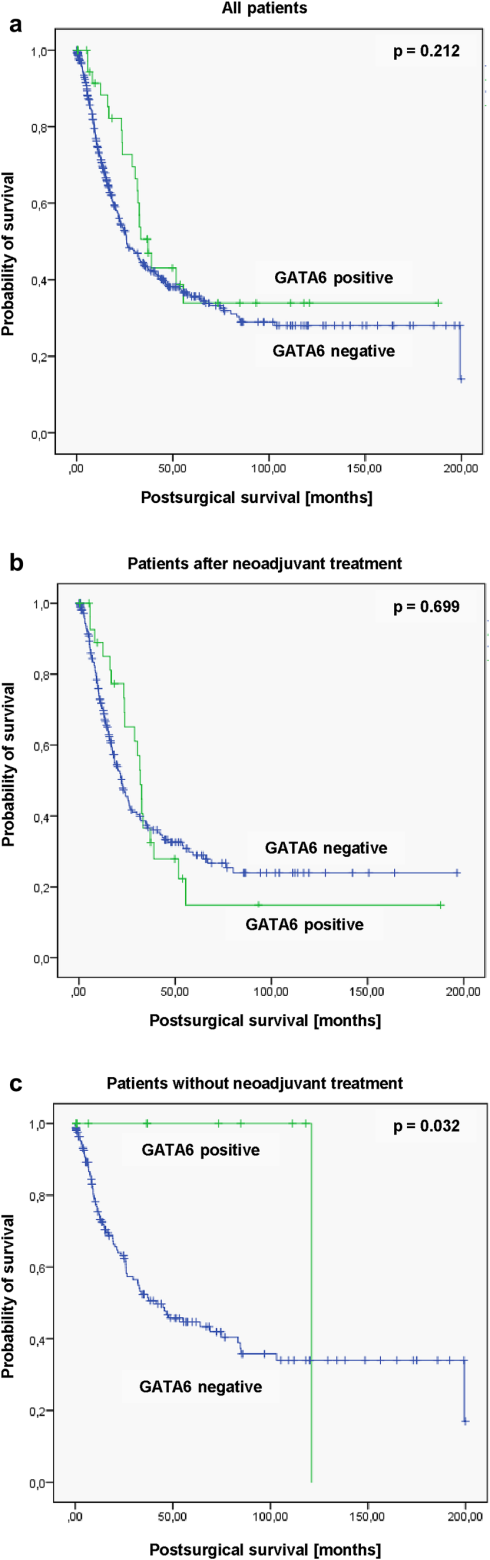
Kaplan–Meier survival analysis (log-rank test) considering the median survival depending on the *GATA6* status of the patients. No significant *GATA6*-depending survival differences were observed within **a** the entire cohort (*p* = 0.212) as well as **b** those patients after neoadjuvant treatment (*p* = 0.699) while the subgroup of *GATA6*-positive patients without neoadjuvant therapy **c** showed a significant better postsurgical survival (*p* = 0.032)

Considering the entire patient cohort of the present study, a significant difference between patients with and without *GATA6* amplification could not be observed (median survival without *GATA6* amplification: 26.1 months (95% CI 20.4–31.7 months) versus median survival with *GATA6* amplification: 37.2 months (95% CI 29.3–45.1 months, *p* = 0.212) (Fig. 2a). The same was true for patients receiving neoadjuvant treatment. In this subgroup, postsurgical survival was comparable between patients with and those without *GATA6* amplification (median survival without *GATA6* amplification: 22.3 months (95% CI 18.2–26.4 months) versus median survival with GATA6 amplification: 31.9 months (95% CI 28.2–35.6 months, *p* = 0.699) (Fig. 2b). However, in patients without neoadjuvant therapy, intratumoral *GATA6* amplification was associated with a prolonged overall survival (OS) compared to those tumors without this amplification (Fig. 2c) (*p* = 0.032). The median OS was 121.1 months (95% CI not calculable) in patients with *GATA6*-amplified tumors in contrast to a median OS of 41.4 months (95% CI 23.4–59.4 months, *p* = 0.032) in patients with normal *GATA6* expression.

Multivariate cox-regression analysis did not confirm *GATA6* as an independent prognostic marker, neither in the entire cohort (*p* = 0.210), nor in the subgroup with (*p* = 0.655) or without neoadjuvant treatment (*p* = 0.961) (compare Table 4 for more details).

**Table 4:** Multivariate cox-regression analysis for all patients and for those with/without neoadjuvant treatment

Factor	All patients				Patients with neoadjuvant treatment				Patients without neoadjuvant treatment			
	Hazard ratio	95% confidence interval		*p* value	Hazard ratio	95% confidence interval		*p* value	Hazard ratio	95% confidence interval		*p* value
		Lower	Upper			Lower	Upper			Lower	Upper	
Sex: male versus female	1.181	0.727	1.918	0.501	1.558	0.836	2.902	0.162	0.481	0.216	1.072	0.074
Age groups: < 65yrs versus > 65 years	1.273	0.980	1.655	0.071	1.170	0.838	1.632	0.357	1.662	1.038	2.662	0.034
Tumor stage:pT1/2 versus pT3/4	1.434	0.992	2.073	0.055	0.874	0.550	1.391	0.571	2.629	1.403	4.924	0.03
Lymph node metastasis: pN0 versus pN +	2.863	2.106	3.981	0.001	2.197	1.504	3.210	0.001	4.294	2.509	7.351	0.001
GATA6:negative versus positive	0.745	0.470	1.181	0.210	0.897	0.558	1.443	0.655	0.001	0.000	10.000	0.961

## Discussion

In the current study, we focused on the frequency and clinical relevance of *GATA6* amplification within a large EAC (*n* = 496) cohort by performing FISH-analysis. We identified gene amplification of GATA*6* in up to 12,6% of patients. However, it had no correlation to clinico-pathological parameters such as sex, age, grading, pT-category, pN-category or UICC-stage. Interestingly, there was a positive correlation between the amplification of *GATA6* and multimodal treatment since patients after neoadjuvant therapy more frequently showed corresponding amplification compared to patients who primarily underwent surgical resection (*p* = 0.044). Additionally, distinct subgroup analysis revealed that an influence of *GATA6* on the patients’ survival was present depending on a multimodal treatment concept. *GATA6* amplification had no effect on the OS in those patients who received neoadjuvant treatment while in patients without neoadjuvant procedures, *GATA6*-positive patients had a significantly prolonged OS. Correlated with other molecular alterations/amplifications common for EAC, we observed a co-amplification of *GATA6* and *PIK3CA* in about 1.8% of patients. This effect was detectable in both subgroups with and without neoadjuvant treatment.

Our current results considering the frequency of amplified *GATA6* is consistent with previous publications by recent large genetic studies (14%) (*n* = 551) (Frankell et al. 2019) or the TCGA-database (12%) (*n* = 185) (compare http://cancergenome.nih.gov/) focusing on this malignancy. Both studies analyze primarily operated tumors (without chemoradiation) and conclude on gene amplification using a next-generation sequencing technique. Using the fluorescence in-situ technique (FISH; gold standard for determining gene amplification) we have the possibility of a direct and reliable visualization of gene copy alterations in tumor cells. In primarily operated tumors we can detect only half of *GATA6*-amplified EACs (6.8%). In our cohort there is an accumulation of *GATA6* amplified tumors in the group of neoadjuvant treated tumors, which has not been considered in all studies so far. However, the vast majority of EACs are now treated neoadjuvantly. Therefore, our results may suggest that *GATA6*-amplified tumors induce an increased resistance to either radiotherapy or chemotherapy.

One study described a much higher frequency of amplification in 20.5% of patients. However, only 85 tumors were included in this work and amplification was observed by performing an array-based comparative genomic hybridization on 20 EACs and further validation via SNP-array analysis and quantitative real-time PCR (qRT-PCR) within the rest of the cohort (Lin et al. 2012). Contrary to this, we performed FISH-analysis which resembles the current gold standard for detection of gene copy number alterations within the daily pathological routine diagnostics.

Although *GATA6* amplification is recurrent in EAC, little is known about the molecular mechanisms this transcriptional factor regulates. *GATA6* amplification increases during the progression from normal esophageal squamous epithelia to Barrett’s metaplasia and finally to the invasive EAC (Pavlov et al. 2015). It was experimentally validated by Van Baal et al. that BMP4, a key protein within the development of Barrett’s esophagus (BE) which induces *SOX9* mRNA expression and which promotor is activated by *GATA6*, is negatively regulated via microRNA (miR)-145 (Van Baal et al. 2013). Overexpression of miR-145 in HET-1A (an esophageal squamous cell line) and BAR-T cells (a non-neoplastic Barrett’s esophagus cell line) resulted in an inhibition of *GATA6*, *BMP4* and *SOX9* expression and in a reduced proliferation rate. This suggested that miRNA-145 might indirectly target *BMP4* via *GATA6* and impact the development of BE (Van Baal et al. 2013). Another in vitro study by Lin et al. demonstrated that ectopic expression of *GATA6* increased anchorage-independent growth in immortalized Barrett’s esophageal cells (Lin et al. 2012). Contrary to this, *GATA6* deprivation induced apoptotic (TNF-associated) pathways in EAC cells (Lin et al. 2012). Own previous data could reveal a possible connection between *Dickkopf-2 (DKK2)* and *GATA6* in EAC (Schiffmann et al. 2020). Nevertheless, it remained unclear how these molecules interact on the molecular level. In pancreatic adenocarcinoma, *GATA6* directly binds to the *DKK2*-promotor leading to a down-regulation of its expression and, therefore, reduces its suppressive effect on the oncogenic Wnt pathways (Zhong et al. 2011). Interestingly, a large genome-wide association study (GWAS) on EAC performed by the German Barrett’s and Esophageal Adenocarcinoma Consortium (BEACON) including about 1065 EAC cases and 1019 controls identified variants of *GATA6* to be strongly associated with the disease reflecting its central role within the tumor development (Becker et al. 2015).

The reasons for the higher frequency of *GATA6* amplification among patients with neoadjuvant therapy in the current analysis are unsolved. It would be interesting to assess putative changes in the *GATA6* amplification rate under therapeutic pressure. To identify dynamic alterations, prospective sample collection of initial treatment-naïve biopsies during the time point of staging followed by consecutive samples from surgical specimens of patients after neoadjuvant therapy would be necessary.

To our best knowledge, this is the first study describing a simultaneous amplification of *GATA6* and *PIK3CA* in EAC. Confirming own previous studies (Schallenberg et al. 2020), no significant co-amplifications with other common CNAs in EAC occurred. We observed amplification of *PIK3CA* in 4.8% of the entire cohort with no differences between patients with or without neoadjuvant treatment as already published by our group (Essakly et al. 2020). However, 1.8% of all patients showed an amplification of both *GATA6* and *PIK3CA*. Chromotrypsis is a recognized oncogenic mechanism of development in EAC. By this route, a synergistic co-amplification of *PIK3CA* and *GATA6* is well conceivable (Nones et al. 2014).

In the present study, we observed a positive prognostic relevance within the subgroup of patients who did not receive neoadjuvant treatment before surgery while the prognosis of the entire cohort was not affected by *GATA6* amplification. After all, the prognosis of EAC patients is still impaired and according to our results upregulation of *GATA6* does not affect this in any manner. On the first sight, this seems contradictory as *GATA6* has been reported to decrease the patients survival in different malignancies (Zhong et al. 2011; Shen et al. 2013, 2019; Tian et al. 2013; Rao et al. 2019). But at second glance the results for EAC are controversial. Some studies with relatively small cohorts of patients (*n* = 73, respectively, *n* = 58) reported poor prognosis in patients with *GATA6* amplifications (Lin et al. 2012; Toxopeus et al. 2019) while another analysis including two separated cohorts (first cohort: 130 tissue samples of normal squamous epithelium, metaplasia, dysplasia, and esophageal adenocarcinoma; second cohort: 92 esophageal adenocarcinoma) demonstrated no association between *GATA6* and overall or disease-free survival in this entity (Pavlov et al. 2015). After all, our own study based on a much larger cohort size utilizing FISH as the gold standard for the detection of copy number alteration in the current pathological routine work-flow. Additionally, there are reports from gastric cancer that suggested multiple roles of *GATA6* within carcinogenesis. Recently a novel suppressive function of *GATA6* has been described within gastric adenocarcinoma revealing that patients with metastatic tumors had low *GATA6* expression with a negative impact on the patients’ survival (Liu et al. 2019). The authors illustrated that *GATA6* directly targets the expression of miR-520b and that this microRNA again reduced its functional target cAMP-responsive element binding protein 1 (*CREB1*) leading to a suppressed cell migration, invasion and metastasis both in *vitro* and in vivo (Liu et al. 2019). Whether these mechanisms are also responsible for the prolonged survival within our study and why this is selectively within those patients with primary surgery remains unclear and needs further investigations.

In summary, our study identified *GATA6* amplification to be significantly associated with multimodal treatment concepts in EAC and to be of prognostic impact for at least those patients with primary surgery. This might indicate an increased resistance to radio-chemotherapy in *GATA6*-amplified tumors. For the first time, simultaneous co-amplification of *GATA6* and *PIK3CA* has been observed within this malignancy. Despite our large cohort, the resulting subgroups for further analysis are quite small (amongst others due to the low frequency of *GATA6* amplification). Consequently, large prospective studies are essential for further validation. Finally, mechanistic approaches for further investigation of the biological functions/interactions related to *GATA6* amplification in EAC via in-vitro, respectively, in vivo experiments should gain more knowledge about how this molecular alteration might be a target for future treatment concepts.

## Data Availability

The datasets generated and/or analyzed during this current study are available from the corresponding author on reasonable request.
